# Genetics and Epigenetics in Obesity: What Do We Know so Far?

**DOI:** 10.1007/s13679-023-00526-z

**Published:** 2023-10-11

**Authors:** Maria Keller, Stina Ingrid Alice Svensson, Kerstin Rohde-Zimmermann, Peter Kovacs, Yvonne Böttcher

**Affiliations:** 1 Medical Department III–Endocrinology, Nephrology, Rheumatology, Medical Center, University of Leipzig, 04103 Leipzig, Germany; 2 Helmholtz Institute for Metabolic, Obesity and Vascular Research (HI-MAG) of the Helmholtz Center Munich at the University of Leipzig, University Hospital Leipzig, 04103 Leipzig, Germany; 3 EpiGen, Department of Clinical Molecular Biology, Institute of Clinical Medicine, University of Oslo, 0316 Oslo, Norway; 4 EpiGen, Medical Division, Akershus University Hospital, 1478 Lørenskog, Norway

**Keywords:** Obesity, Genetic variants, Epigenetic marks, Polygenic risk scores, Methylation risk scores

## Abstract

**Purpose of Review:**

Enormous progress has been made in understanding the genetic architecture of obesity and the correlation of epigenetic marks with obesity and related traits. This review highlights current research and its challenges in genetics and epigenetics of obesity.

**Recent Findings:**

Recent progress in genetics of polygenic traits, particularly represented by genome-wide association studies, led to the discovery of hundreds of genetic variants associated with obesity, which allows constructing polygenic risk scores (PGS). In addition, epigenome-wide association studies helped identifying novel targets and methylation sites being important in the pathophysiology of obesity and which are essential for the generation of methylation risk scores (MRS). Despite their great potential for predicting the individual risk for obesity, the use of PGS and MRS remains challenging.

**Summary:**

Future research will likely discover more loci being involved in obesity, which will contribute to better understanding of the complex etiology of human obesity. The ultimate goal from a clinical perspective will be generating highly robust and accurate prediction scores allowing clinicians to predict obesity as well as individual responses to body weight loss-specific life-style interventions.

**Supplementary Information:**

The online version contains supplementary material available at 10.1007/s13679-023-00526-z.

## Introduction

Obesity rates are steadily increasing [1] and represent a major public health thread worldwide. Being an important cause for concomitant metabolic co-morbidities such as type 2 diabetes, dyslipidemia, cardiometabolic diseases including coronary artery diseases, stroke and hypertension as well as for some types of cancers [2], obesity substantially reduces life expectancy [3]. As summarised by the World Obesity Atlas 2023 [4], about 988 million people (aged > 5 years) worldwide were affected with obesity (BMI ≥ 30 kg/m^2^) in 2020, which is estimated to dramatically increase by 2035 to 1.914 billion. This corresponds to a proportional increase of the population with obesity from 14% in 2020 to 24% in 2035, clearly illustrating the need to prevent and treat obesity.

Obesity is a multifactorial disease being governed by both genetics and environmental factors originating from a rather “obesogenic environment” such as sedentary lifestyle with reduced energy expenditure and high calorie diet intake. The existence of a genetic background in obesity is undisputable and first evidence was provided by family [5–8], twin [9–11] and adoption [12] studies that have clearly estimated heritability rates for BMI between 40 and 70%. Genome-wide association studies (GWAS) have to a large extent contributed to an improved understanding of the genetic architecture of common obesity and have provided hundreds of novel risk variants [13–15]. However, although significant advances have been made in describing the mechanistic circuitry for a least some of these genetic variants [16, 17], identifying novel risk variants in general precedes the biological and functional understanding of how these variants act in a certain target tissue in order to increase body weight. Furthermore, the variability of BMI attributed to genetic variation is still poorly explained [15]. The major challenge here is a combination of genetics with environmental factors such as energy intake, physical activity, smoking, but also gene–gene interactions. These interactions may introduce additional inter-individual variability, illustrating the highly dynamic and complex etiology underlying the pathophysiology of obesity.

Epigenetic analyses have therefore been largely accelerated during the last years with epigenome-wide association studies (EWAS) dominating the field. Numerous genes and novel CpG sites were identified conferring changes in methylation profiles in obesity [18]. However, causal interferences in obesity are still under debate, yet a few studies implicate a causal role of obesity in inducing changes in methylation levels [19•, 20].

To translate the bench-side generated knowledge into a clinical day life and to generate a useful tool helping to predict obesity (e.g. based on BMI changes), significant effort was put in designing polygenic risk scores and more recently, also methylation risk scores. These scores represent a weighted combination of several genetic variants or methylated CpG sites at many different positions across the human genome. However, so far, the use of such scores is rather limited as reliable prediction is not yet possible or to a substantial part inaccurate. Taken together, enormous progress has been made in understanding the genetic architecture of obesity and the correlation of epigenetic marks with obesity and related traits. This review aims at highlighting current research and its challenges in genetics and epigenetics of obesity.

## Genetic Background of Common Polygenic Obesity

Lessons from monogenic obesity have significantly contributed to our general knowledge on genetics and physiology of body weight regulation. However, non-syndromic monogenic obesity affects only about 5% of the population with obesity [21]. About 95% of the individuals with obesity develop common polygenic obesity, which is multifactorial and assessing the heritability of polygenic obesity is still one of the major challenges, despite recent advances in genetics of obesity. Genome-wide strategies including linkage and genome-wide association studies (GWAS), which are hypothesis-free per se have been of paramount importance in discovering novel genes involved in the complex etiology of human obesity.

### Identifying Novel Genetic Markers by Using Genome Wide Approaches—Linkage Analyses

Genome-wide linkage analyses allow testing for co-segregation of polymorphic genetic markers with phenotypic traits/disease in families, trios or sibling studies. The approach proved to be enormously efficient in discovering genetic variants in monogenic forms of obesity. However, when employed to discovery efforts for underlying genetic markers in polygenic forms of obesity, linkage analyses had only a marginal impact, as most of the identified susceptibility loci for obesity could not be replicated and confirmed in subsequent studies or fine mapped to identify the causal variants affecting the disease. This is most likely to be attributed to small sample sizes in the performed linkage analyses as well as to the poor coverage of genetic variation in tested genomes. One of the very few promising genes discovered in a linkage study was the *ectonucleotide pyrophosphatase/phosphodiesterase 1 gene* (*ENPP1*), located on chromosome 6q. The gene was initially discovered to be related to childhood obesity and associated traits by genome-wide linkage analyses [22] and one of its haplotypes further replicated in independent childhood cohorts as well as adults [23, 24]. It is of note however, that despite some inconsistencies in replication efforts, a large meta-analysis including 24,324 individuals clearly supported the potential role of the *ENPP1* Q121 variant in the pathophysiology of obesity [25].

### Identifying Novel Genetic Markers by Using Genome Wide Approaches—GWA Studies

Whilst the above-described approaches like candidate gene and genome-wide linkage studies showed only marginal success in discoveries of susceptibility genes for common polygenic obesity, prominent advances in molecular biology, including high-throughput genotyping techniques, have enabled researchers to use GWAS to identify novel genetic loci associated with human obesity. This has indeed led to a dramatic increase of until then unknown genetic variants associated with obesity. Started with the discovery of genetic variants in the *fat mass and obesity-associated gene* (*FTO*) reported in 2007 [26, 27], so far, more than 1000 loci carrying variants including single nucleotide polymorphisms (SNPs) significantly associated with measures of obesity like BMI have been identified in meta-analyses of large-scale GWAS. These efforts were mostly coordinated within international consortia such as GIANT (the Genetic Investigation of ANthropometric Traits) [13, 15], which predominantly included populations of European ancestry. However, a number of well-powered studies including populations of Asian [28, 29], Hispanic [30] and African [31] ancestries contributed to new discoveries or replication of already reported obesity susceptibility loci. These populations helped to increase the size of available cohorts and so the statistical power of the GWAS. Moreover, based on their specific demographic and evolutionary characteristics, they were particularly valuable in identifying genetic variants with larger effect sizes specific for the respective population. One of these ethnic groups is the Greenlandic population, which played a crucial role in identification of obesity-associated polymorphisms in *ADCY3* [32, 33], a gene which may play a role in the regulation of human body weight [34].

**Fig. 1:**
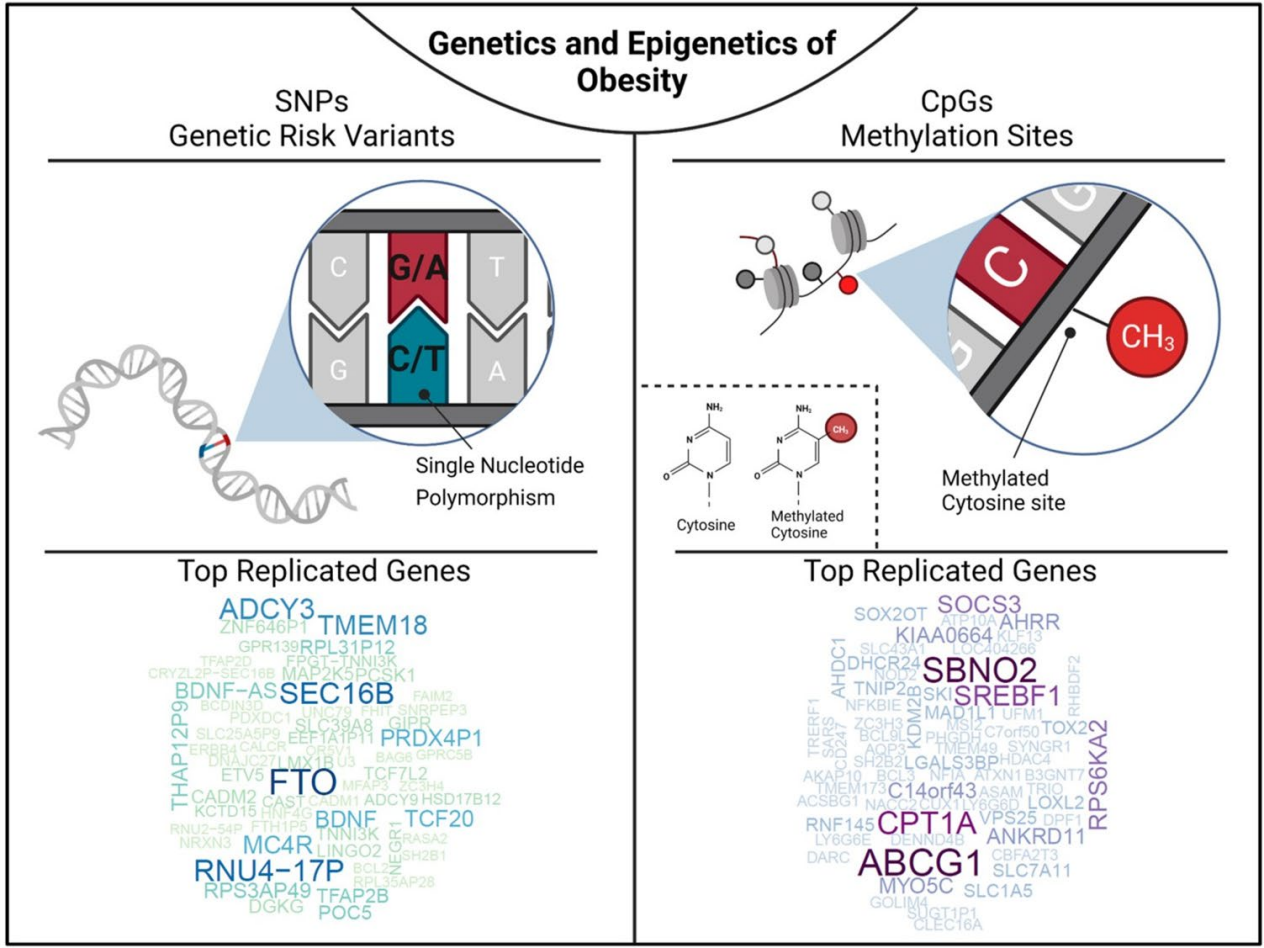
Genetics and epigenetics of obesity. The figure illustrates that single nucleotide polymorphisms are genetic risk variants identified by GWAS and CpG sites being differentially methylated in obesity. To screen for the most frequently replicated genes close to identified SNPs and CpGs for associations with BMI, we accessed the GWAS (BMI in adults and children) and EWAS catalogue (BMI in adults), respectively. SNP and CpG associations with more than one annotation were handled as individual gene count. Only hits with a *P* < 1 × 10^−8^ were included. Associations were analysed for replication frequencies and blotted using the wordcloud package in R (version 4.2.0, https://blog.fellstat.com/?cat=11). Most replicated GWAS hits for BMI: Word cloud presenting the most often replicated gene hits for genome-wide association of SNPs with BMI in adults and children (GWAS catalogue accessed 20.03.2023 [44]). All genes are replicated at least fifteen times. Gene name size and colour intensity (light green to dark blue) are indicating the replication strength (from least [15 times] to most [56 times]). Long-non-coding RNAs were excluded. Most replicated EWAS hits for BMI: Word cloud presenting the most often replicated gene hits for epigenome-wide association studies with site-specific DNA methylation marks for BMI in adults (EWAS catalogue accessed (08.03.2023 [18]). All genes are replicated at least three times. Gene name size and colour intensity (light blue to dark purple) are indicating the replication strength (from least [three times] to most [nine times]). The upper panel of the figure was generated by using BioRender.com

The GWAS findings indicate that even with hundreds of obesity-associated loci identified to date, they only explain about 6% of the variation of BMI [15]. Although the remaining variability of BMI remains one of the major challenges of the future research efforts, genome-wide strategies have clearly demonstrated their enormous potential in discovering novel disease susceptibility loci (Fig. 1). In the context of obesity, they showed that most of the identified loci harbour genes involved in pathways affecting neuro-circuits of appetite and satiety regulation (*BDNF*, *MC4R* and *NEGR*) [35–37], energy and lipid metabolism (*FTO*, *RPTOR* and *MAP2K5* [13, 27, 38], insulin secretion and action (*TCF7L2*, *IRS1*) [13, 38] as well as adipogenesis [14]. Furthermore, GWAS also suggested that many of the identified obesity-associated genes are common also for other metabolic diseases such as diabetes, hypertension, and coronary artery disease, which has been supported in gene ontology analyses (GO) highlighting gene clusters with common shared metabolic pathways for these diseases [39]. Another important takeaway from GWAS is the fact that numerous common polymorphisms associated with polygenic obesity in ethnically diverse population have been found in genes like *PCSK1* [40–42], *MC4R* [43••] and *POMC*, known to carry rare loss of function variants leading to non-syndromic monogenic obesity. Although the GWAS are an excellent tool to uncover variants associated with complex non-Mendelian traits and diseases, understanding the underlying mechanisms behind these associations remains challenging. The majority of genetic variants associated with obesity map within non-coding regions without any obvious biological function, may however carry regulatory elements essential in molecular processes such as gene regulation. Finding the respective target gene of the associated variants appears often difficult since they may be located in distant chromosomal regions, which need to be assessed in subsequent follow up studies. For instance, despite the relatively large effect of the *FTO* SNPs on BMI with 0.35 kg/m^2^ per allele or 1 kg for a person who is 1.7 m tall reported in 2007 [27], it took until 2014 to explain the regulatory circuitry and mechanistic chains behind the associations between *FTO* variants and obesity. Claussnitzer et al. not only showed that the intronic BMI-associated *FTO* SNP maps within an enhancer element for ARID5B, but could also demonstrate that ARID5B regulates the expression of *IRX3* and *IRX5*, which finally affect adipogenesis, lipid accumulation and thermogenesis [16].This study impressively demonstrated that comprehensive and well-designed functional studies are essential to elucidate molecular pathways underlying the observed associations of genetic loci with obesity.

### Genome-Wide Association Studies and Polygenic Risk Scores in Children

The major part of research efforts in polygenic obesity has been focused on adult cohorts [45], whereas similar studies in childhood obesity are rather sparse [46] and are mainly concentrating on replication of findings achieved in adults. It is of note however, that most of the loci identified in adults also associate with obesity in children suggesting the impact of genetic variants across the entire lifespan [47, 48]. Exemplarily, polymorphisms in *FTO* and *MC4R* have been shown to be significantly associated with childhood and adolescent obesity in populations from diverse ethnic backgrounds [49–56]. Nevertheless, effects of some SNPs appear to be more pronounced in children and diminish later in life as has been shown for the associations of variants in *TMEM18*, *GNDPA2*, *MC4R*, *NEGR1*, *BDNF* and *KTCD15* with early-onset obesity [57], and particularly for *INSIG2* variants [58–60]. Interestingly, some studies reported that diabetes susceptibility alleles in the HHEX-IDE locus were associated with increased BMI in children, which may underpin the well-acknowledged association between childhood obesity and T2D later in adults [61].

In the context of childhood obesity, polygenic risk scores (PGS), which represent a simple model to determine genetic risk based on multiple genetic variants at different positions in the genome, may render an important tool in translation towards precision medicine. PGSs calculated in early life would enable detection and stratification of individuals with different degrees of obesity risk, and thus, the specific time windows for targeted individualised therapies could be developed [62]. Unfortunately, to date, PGSs are mainly calculated from GWAS performed in adults, which might cast doubts on their informative value for paediatrics. However, as shown by Khera et al. (2019), these doubts do not seem to be justified by the recently generated data [63••]. Here, the authors successfully demonstrated that a polygenic predictor based on 2.1 million known obesity variants is not only associated with a 13 kg increase in body weight in adulthood, but also at birth (+0.06 kg) and at 8 years of age (+3.5 kg) [63••]. Moreover, this study indicated that PGSs derived from adult data may have a comparable strong association with BMI in children. The predictive potential of PGSs in discriminating weight differences in this study was promising and could even be further refined by considering other non-genetic factors such as maternal BMI [64]. However, using this PGS in order to predict future obesity in the UK Biobank has been rather disappointing, showing a high proportion of unreliability making it less useful in clinical utility regarding disease prediction [45]. This clearly sheds light on the difficulties in using genetic information for common polygenic obesity and to translate it into a clinical prediction tool that can be a game changer in clinical day life and decision making. This is currently unrealistic although, it seems conceivable that the performance of PGSs can be increased by combining it with other factors such as environmental or epigenetic indicators to function more accurately [45]. Indeed, it is well-acknowledged that genetic profiling is gaining general popularity in extensive research endeavours, such as within large-scale biobanks linked to healthcare and clinical trials. As a result, it is more and more common for patients and their doctors to encounter PRS during clinical interactions, such as those related, e.g. to cardiovascular conditions [65], severe liver disease [66] and other human pathologies. Thus, refined and robust scores providing more accurate prediction of obesity in the future will undoubtedly become important measures in clinical settings potentially conferring also a predictive value for risk of developing obesity-related co-morbidities such as cardiovascular diseases, liver disease and several types of cancers.

## The Importance of Epigenetic Mechanisms

In the context of the complex etiology of human obesity, epigenetic mechanisms based on, e.g. DNA methylation or histone modifications and gene-environment interactions are important to be considered in order to better understand the role of genetics in the development of this multifactorial disease. Despite large-scale GWAS and a simultaneously rising number of studies addressing gene-environment interactions, these studies remain challenging and their findings are often population-specific and not ubiquitously applicable and straightforward. Environmental factors such as physical activity, smoking and dietary components are acting as modifiers of the genetic predisposition to obesity manifestation. This clearly highlights obesity as a preventable disease and further indicates a highly beneficial potential of treatment strategies based on lifestyle interventions. A recent review reported the majority of SNP-environment interactions in association with alcohol consumption, smoking and physical activity [67]. However, among them were also robustly replicated associations as reported for the *FTO* locus which effects could be attenuated by increased physical activity, but exaggerated by non-healthy fried food consumption [68–70]. There is no doubt that the steadily increasing number of large-scale studies including cohorts such as the UK Biobank and similar large-scale efforts will lead to the discovery of new and more robust gene-environment interactions in the future, usable for more precise treatment opportunities. Nevertheless, the underlying causative mechanisms behind the observed associations remain unknown for most of the genes.

### Epigenome-Wide Association Studies in Obesity

Epigenetic mechanisms such as DNA methylation or modification of histone core proteins are suggested to mediate gene-environment interactions and therefore may play a substantial role in susceptibility for obesity. DNA methylation is the most stable, easy to measure and best studied epigenetic mark and has been extensively studied over the last years in relation to obesity.

Genome wide DNA methylation patterns are widely used for EWAS aiming to uncover DNA methylation marks correlating with clinical variables of obesity or fat distribution. Thus, a rapid rise of well-powered EWAS (including multi-omics approaches) and partly large case–control studies in twins, family settings or independent subjects started almost one decade ago and discovered novel targets being involved in epigenetic dysregulation in obesity. In the present review, we summarised 45 genome-wide methylation studies including work mostly conducted in Caucasian subjects and performed in DNA samples originating from whole blood, isolated blood cells or adipose tissue, (Table 1). Importantly, although DNA methylation analyses truly identify novel candidate CpG sites and genes, additional information such as on genetic variation, gene expression, proteome/metabolome is warranted to understand the causative mechanistic circuitry underlying the correlation with disease relevant clinical traits. For instance, genetic variants may modulate the methylation at specific CpG sites potentially inducing co-methylation patterns at nearby sites, eventually translating into changes in clinical traits and suggesting a genotype–phenotype correlation. Therefore, a rising number of studies focus on multi-omics epigenetic associations with obesity or related traits, mainly promoted by the latest advances in high-throughput technologies and analytical approaches promoting (Table 1).

**Table 1:** Epigenome wide Association Studies for BMI and related clinical traits of obesity

Study reference	PMID	Clinical trait	Ethnicity	*N*/females; age in years	Tissue	Main findings	Multi-omics	Meta-analysis
Carless et al. (2013) [71]	24,058,506	WC, BMI	Mexican American	183/101 19–75	Blood	Genetic effects on DNA methylation, no significant associations to obesity measures		
Xu et al. (2013) [72]	23,644,594	BMI	African American	96/48 14–20	Blood	Methylation variability in obesity		
Dick et al. (2014) [73]	24,630,777	BMI	Caucasian	459/84 ø55	Blood, adipose tissue	Methylation of HIF3A associated with BMI		
Almén et al. (2014) [74]	25,010,727	BMI	Caucasian (Latvia)	46/46 41–70	Blood	Age-associated epigenetic changes are influenced by obesity		
Guenard et al. (2014) [75]	24,495,915	MetS	Caucasian (American)	14/0 ø38	Adipose tissue	Multiple novel targets, pathways are related to cell membrane, inflammation; immunity; cell cycle		
Aslibekyan et al. (2015) [76]	26,110,892	WC, BMI	Caucasian (European American)	991/515 ø49	CD4 + T-cells	DNA methylation of *CPT1A*, *PHGHD*, *CD38* and lncRNA00263 BMI/WC		
Ollikainen et al. (2015) [77]	25,866,590	BMI	Caucasian	80/46 ø27	Blood	Differences in DNA methylation due to excessive liver fat		
Demerath et al. (2015) [78]	25,935,004	WC, BMI	African American	2097/1334 47–70	Blood	164 and 8 probes for WC and BMI respectively, including *HIF3A*, *CPT1A* and* ABCG1*		
Voisin et al. (2015) [79]	26,449,484	obesity	Caucasian	355/141 14–34	Blood	28 obesity SNPs associate with 107 proximal CpG site in obesity-related genes	Methylome, genotype	
Arner et al. (2015) [80]	26,351,548	BMI	Caucasian	29/29 ø46	Adipose tissue	5529 differentially meth CpG sites correlate to 2223 diff expressed genes being related to metabolic function of fat cells	Methylome, transcriptome	
Rönn et al. (2015) [81]	25,861,810	BMI, HbA1c, age	Caucasian	190/94 23–80	Blood, adipose tissue	DNA methylation and expression of 2825 genes correlated with BMI, of 1050 genes with age and 711 CpG site with HbA1c	Methylome, transcriptome	
Kirchner et al. (2016) [82]	26,977,391	BMI, T2D	Caucasian	35/0 21–62	Liver	hypomethylated genes (ATF-motifs) in the liver of obese subjects independent of T2D	Methylome, transcriptome	
Ali et al. (2016) [83]	27,564,309	BMI, MetS	Caucasian	192/106 8–90	Blood	Identification of *SOCS3* methylation being associated with BMI, MetS and related lipid traits		
Keller et al. (2016) [84]	28,123,940	BMI	Caucasian	105/66 ø57	Adipose tissue	Identification of six robust adipose tissue depot-specific genes (*HAND2*, *HOXC6*, *PPARG*, *SORBS2*, *CD36*, and *CLDN1*)	Methylome, transcriptome	
Pietiläinen et al. (2016) [85]	26,499,446	BMI	Caucasian	74/42 23–36	Adipose tissue	Diff methylated and expressed genes in monozygotic twins discordant for BMI, suggesting pathological adaptation of SAT to obesity is partly epigenetically regulated	Methylome, transcriptome	
Volkov et al. (2016) [86]	27,322,064		Caucasian	119/ 22–80	Adipose tissue	Discovered mQTLs including *ADCY3/POMC*, *APOA5*, *CETP*, *FADS2*, *GCKR*, *SORT1* and *LEPR*. 635 SNPs in mQTLs associated with expression of 86 genes	Methylome, genotype, transcriptome	
Al Muftah et al. (2016) [87]	26,823,690	BMI, T2D	Arabs	123/72 ø38	Blood	replicated eight CpG site associations in Arabs; suggest heterogeneity of effects underlies genetic variation		
Sayols-Baixeras et al. (2017) [88]	29,099,282	WC, BMI	Caucasian	641/325 35–79	Blood	Discovery of 70 and 30 novel CpGs associated with BMI and WC, respectively, explaining 25.94% and 29.22% of the variability of BMI and waist circumference		yes
Crujeiras et al. (2017) [89]	28,211,912	obesity	Caucasian	55/28 20–83	Blood, adipose tissue	*FGFRL1*, *NCAPH2*, *PNKD* and *SMAD3* diff methylated between obese and non-obese subjects		
Wahl et al. (2017) [20]	28,002,404	BMI	Caucasian, Indian Asian	5387/2147 ø54		EWAS for BMI identified 187 genetic loci differentially methylated; the study demonstrated that epigenetic changes are likely the consequence of obesity rather that the cause		
Meeks et al. (2017) [90]	28,947,923	WC, BMI, obesity	Sub-Saharan Africans	547/316 ø50	Blood	The first EWAS for obesity in Africans identified three epigenome-wide significant loci (*CPT1A*, *NLRC5* and *BCAT1*)		
Wilson et al. (2017) [91]	27,773,939	BMI	Caucasian (North American)	871/871 ø55	Blood	Identified CpG sites at *ANGPT4*, *RORC*, *SOCS3*, *LGALS3BP* associated with BMI		
Guenard et al. (2017) [92]	28,219,716	MetS	Caucasian (North American)	31/0 ø35	Blood, adipose tissue	Identified 2182 meQTLs regulating the methylation levels of 174 CpG sites; revealed *COL11A2* meQTLs corr with fasting glucose levels	Methylome, genotype	
Mendelson et al. (2017) [93]	28,095,459	BMI	Caucasian	3743/1947 ø72	Blood	The study discovered novel and replicated known BMI-associated at 83 CpGs loci	Methylome, transcriptome	
Kvaløy et al. (2018) [94]	30,397,228	BMI	Caucasian	120/120 23–31	Blood	Identified 10 diff meth CpG sites: *COX6A1P2/FGD2*, *SBNO2*, *TEX41*, *RPS6KA2*, *IGHE/IGHG1/IGHD*, *DMAP1*, *SOCS3* and* SETBP1*		
Dhana et al. (2018) [95]	29,762,635	WC, BMI	Caucasian	1450/811 ø64	Blood	Discovered 12 and 13 CpGs associated with BMI and WC, respectively. The most significant CpGs were annotated to *MSI2* and *LARS2*		
Campanella et al. (2018) [96]	29,713,043	WC, BMI, WHR, WHeR	Caucasian	1941/1353 ø54	Blood	40 CpG loci were associated with at least one adiposity measure. One CpG at *ABCG1* was associated with all four traits other clinical variables	Methylome, transcriptome	Yes
Orozco et al. (2018) [97]	29,566,149	MetS	Caucasian	201/0 45–73	Adipose tissue	Identification of 18 candidate genes, including known and novel genes. Methylation deconvolution demonstrated the loci were specific to adipocytes	Methylome, transcriptome	
Akinyemiju et al. (2018) [98]	29,643,945	MetS	African American	614/411 ø49	Blood	Two differentially methylated CpGs annotated to *IGF2BP1* and *ABCG1* were identified, *ABCG1* could be replicated		Yes
Zaghlool et al. (2018) [99]	29,325,019	BMI	Arab Asians, Filipinos	359/177 ø47	Blood	Reports potential causal effects of metabolite levels on methylation of obesity-associated CpG sites, such as on *DHCR24*, *MYO5C* and* CPT1A*	Methylome, proteome, metabolome	
Guo et al. (2018) [100]	30,619,480	BMI	Caucasian, Asian,African American, European American	22,310/n.a n.a	Blood, adipose tissue	119 CpGs associated with obesity were identified, *SOCS3* was part of enriched pathways and differentially expressed in adipose tissue	Methylome, transcriptome	Yes
Wang et al. (2018) [25]	29,312,471	BMI	African American	700/n.a 14–36	Blood	The study identified 76 obesity-related CpG sites; 54 were replicated	Methylome, transcriptome	
Li et al. (2019) [101]	31,152,155	BMI	Asian	60/30 39–72	Blood	The study identify 30 CpGs; however, none of the sites reached genome-wide significance. 11 differentially methylated regions were validated	Methylome, transcriptome	
Li et al. (2019) [102]	31,480,455	BMI	African American	232/232 ø32	Saliva	The study suggests that high BMI accelerates DNA methylation age		
Pan et al. (2019) [103]	30,837,522	BMI	African American, European American	24/0 14–20	Blood	The group identified a novel neutrophil activation *ALPL* in obesity *ALPL* expression associates with CVD risk factors	Methylome, transcriptome, proteome	
Koh et al. (2020) [104]	32,788,176	BMI	Asian	450/0 ø47	Blood, adipose tissue	The study discovered multiple diff meth CpG sites and altered gene expression, suggesting *CPA3* as potential obesity-related gene	Methylome, transcriptome	
Giri et al. (2020) [105]	32,363,570	BMI	Indo-European	1142/545 Ø50	Blood	The study discovered genetic markers in *SLC22A11* and *BAI3* that associated with DNA methylation at important cis-regulatory elements	Methylome, genotype	
Justice et al. (2020) [106]	32,901,515	BMI	African American	2684/1702 45–64	Blood	Identified novel CpG sites near *TXNIP*, *ADCY7*, *SREBF1* and *RAP1GAP2* that were not previously described for obesity		
Xie et al. (2021) [107]	34,556,110	WC, WHR	Caucasian	210/105 ø28	Blood	This work idenfied a CpG site cg16170243 significantly associated with BMI adjusted WC		
Chen et al. (2021) [108•]	34,670,603	WC, BMI	Asian (multi-ethnic: Chinese, Malay, Indian)	409/338 ø51	Blood	Discovery of multiple CpG sites associated with BMI and WC. Analyses suggest high BMI is rather cause than consequence of changes in DNA methylation		
Cao et al. (2021) [109]	33,810,959	WC, BMI, HC, WHR	Norfolk Island isolate	47/24 ø42	Blood	Multi-trait analysis based PCA identifies two PC components explaining ~ 89% of the phenotypic variance; identified 5 CpGs at *GOT2-CDH8*, *LYSMD3*, *HIBADH*, *ADGRD1* and *EBF4* genes		
Do et al. (2021) [110]	34,278,703	WC, BMI	Caucasian (European American)	43,936/n.a 18–75	Blood and other tissues	The study discovered 52 CpG sites associated with BMI being potential mediators of obesity-associated chronic diseases		Yes
Wu et al. (2022) [111]	35,882,828	WHR	Northern Han Chinease	120/n.a ø52	Blood	The study identified numerous CpG sites whose methylation levels associate with WHR		
Taylor et al. (2023) [112]	36,479,596	BMI	African American	239/239 ø31	Saliva, blood	A combined sex and female-only meta-analyses discovered multiple CpG sites associated with BMI		Yes
Do et al. (2023) [113•]	36,649,705	BMI	Caucasian/European, Asian, African	17,034/n.a n.a	Blood	This study identified ~ 700 novel CpG sites associated with BMI and that 397 CpG sites explained 32% of the variance in BMI		Yes

This table summarises 45 studies identified by using the following criteria: We performed a PubMed search (dated 08/03/2023) for studies published during the last 10 years (2013–2023) using the following mesh-terms: DNA methylation AND obesity/EWAS AND obesity. We focused on studies analysing genome-wide DNA methylation by individual techniques (arrays, WGBS) associated with BMI or other obesity-related traits. We excluded longitudinal studies measuring DNA methylation changes induced by different types of interventions, studies mainly focusing on T2D, cancer, transgenerational effects or prediction models, studies only performed in vitro or other than human model organisms. In addition, we focused on DNA methylation only and excluded other epigenetic mechanisms such as histone modifications and non-coding RNAs. The table further provides information about the analysed trait(s) (BMI; *WC* waist circumference, *WHR* waist-to-hip ratio, *WHeR* waist-height-ratio, *T2D* type 2 diabetes, *MetS* metabolic syndrome etc.), ethnicity, sample size (discovery cohort), number of females included, age range or mean age (ø), tissue of origin, information about the reference (PMID), publication year and the major results. We further indicated studies that have analysed two or multi-omics levels (methylome, genotype, transcriptome, proteome and metabolome) and those using meta-analyses strategies. Here, we count a study to be “multi”-omics if the authors analysed more than one level of the omics cascade

Although the most powerful EWAS reported recognisable sample sizes with more than 5000 subjects in the discovery cohort [20], the effect sizes are highly variable ranging from 6 to 40 kg/m^2^ change in BMI per unit increase in blood DNA methylation. In general, sample sizes in EWAS studies are often much smaller than in GWAS analyses (Table 1), with the smaller cohorts mainly estimating methylation differences between individuals with and without obesity/metabolic syndrome. Studies with lower sample sizes also report smaller effect sizes such as 0.8–3.6% BMI increase per 0.1 increase in methylation ß-values [73]. Interestingly, Vehmeijer et al. demonstrated an increasing effect size with age by meta-analysing 187 methylation loci, previously reported to show cross-sectional association to BMI in adults, in children with an age between 2 and 18 years [114]. However, most studies are still of explorative nature focussing on the identification of novel candidate sites and genes rather than evaluating whether methylation changes are cause or consequence of obesity (Table 1).

To generate an overview about genes reported to show associations between methylation level of specific CpG sites and BMI, we used data from the EWAS catalogue (all *P* < 1 × 10^−8^; EWAS catalogue accessed 08.03.2023, [18]) and performed a word-cloud analysis using gene IDs in R (*wordcloud* package, R version 4.2.0). Based on this analysis, we estimated the most replicated genes originating from EWAS for BMI (Fig. 1). All genes included were replicated at least in three studies with *ABCG1* (*ATP-binding Cassette Sub-family G Member 1*) being the mostly replicated gene locus followed by *CPT1A* (*Carnitin Palmitoyltransferase 1*), *SREBF1* (*Sterol Regulatory Element Binding Transcription Factor 1*), *SBNO2* (*Strawberry Notch Homolog 2*) and *SOCS3* (*Suppressor of Cytokine Signaling 3*). Among them *ABCG1* [78, 91, 96, 98] and *CPT1A* [76, 78, 87, 90, 99] were described across different ethnicities such as Caucasian, African American, Africans and Asians (Table 1). Of note, some larger cohorts such as LOLIPOP (London Life Sciences Prospective Population [115]) or KORA (Cooperative Health Research in the Region of Augsburg [116]) are more frequently used as replication cohorts.

Several studies support the functional role of, e.g. *ABCG1* and *CPT1A* in obesity. Wahl et al. [20] for instance reported an association of the BMI genetic risk score with the *ABCG1* methylation being consistent with other studies reporting effects of overweight and weight-loss on methylation, expression or protein activity [20, 117, 118]. In general, ABCG1 is involved in mitochondrial cholesterol efflux, thus promoting cellular efflux to HDL. Its silencing leads to massive lipid accumulation in tissues of high fat and high cholesterol fed mice and in 3T3L1 adipocytes [119–121]. In line with this, *ABCG1* and, e.g. *SREBF1* methylation levels are also known to correlate with T2D [115, 122–124], postulating direct or indirect effects on metabolic consequences of obesity. Similar to ABCG1, CPT1A is involved in mitochondrial fatty acid oxidation and ROS production by regulating the entry of long-chain fatty acids into the mitochondrial matrix [125] and thereby also contributing to the activation of inflammasomes [126]. Furthermore, high-fat diet (+/− fructose) fed mice revealed a decreased CTP1a activity and thus a decreased fat metabolism, whereas knockdown of the fructose metabolism enhanced CPT1a activity [127]. Taken together, EWAS studies have extensively helped to discover CpG sites whose differential methylation levels correlate with important clinical traits of obesity and fat distribution, thus clearly illustrating the importance of epigenetic marks in obesity and its potential dysregulation in disease. However, despite these efforts and multiple novel candidate genes identified during the last years, the precise mechanistic circuitry of those genes in the human pathophysiology of obesity and relevant metabolic traits is still not well understood. Furthermore, although recent studies support the role of methylation changes in obesity, to what extent whole blood methylation profiles can mirror their patterns in target tissues remains under discussion. In addition, the majority of studies included in this review used array based approaches for genome-wide association studies, providing a limited overview of 1.7–3% of all CpG positions in the genome, illustrating that a large part of the remaining sites is undiscovered among these studies [128].

### Ethnicity Specific Findings and Meta-analyses

Although most genome wide DNA methylation analyses were conducted in cohorts with Caucasian ancestry (Table 1), recent studies focussed more on the homo- or heterogeneity between the ethnic groups. For instance, a EWAS performed in an Arab population confirmed seven previously identified BMI loci but reported higher effect sizes compared to their replication cohort from the UK [87]. However, it has to be acknowledged that the reported association of these loci did not reach genome-wide significance level in the Arab discovery cohort. In line with this, previously reported associations between, e.g. *CPT1A* methylation and BMI have been confirmed across multiple ethnicities such as Caucasian, African American, African, Asian and Arab [76, 78, 87, 90, 99]. Moreover, a recently published multi-ethnic study in Asians was able to replicate 110 BMI-associated loci, which were previously reported for Europeans, South Asians and African Americans with a high consistency of the effect directions. Although they reported a great homogeneity across the different Asian ethnicities, they also demonstrated heterogeneity across several loci, where for instance the effects are mainly driven by the Chinese subjects [108•]. Another study, taking into account a longitudinal setting, discovered a total of 287 novel CpG sites correlating with BMI (266 in white participants, 21 in black individuals). Importantly, a major take home message from this report is that, based on the longitudinal design, the authors concluded that obesity seems to precede changes in methylation, underlining, in line with Wahl et al. [20] that obesity may rather be cause than consequence of epigenetic changes [19•].

In a very recent study, representing the largest meta-analysis so far, Do et al. [113•] performed an EWAS in more than 17,000 individuals to detect CpG sites associated with BMI of European (Caucasian), African and Asian subjects. Following this approach, the study confirmed 553 previously reported loci but also identified 685 novel sites, which were successfully replicated. Interestingly, only five CpG sites were reported showing an interaction with BMI by race/ethnicity among individuals with a European or African ancestry. Importantly, in an attempt to assess the value of such CpG sites in predicting BMI, the study demonstrated that 397 of those identified CpG positions explained 32% of BMI variability illustrating that a methylome-based prediction of BMI in this study performed relatively good [113•].

### The Utility of Methylation Risk Scores in Predicting Disease Risk

Similar to genetic analyses, the concept of polygenic risks scores can be transferred to CpG methylation data and can be used to construct methylation risk scores (MRS). Such MRSs may prove useful tools in predicting disease risk or assessing exposure to specific environmental factors in the future. It is noteworthy, however, that in addition to methodological challenges in constructing weighted MRSs, all EWAS findings, and thereby MRSs are highly sensitive to potential confounders such as age, gender, ethnicity and also technological differences in assessing DNA methylation [129]. In line with Do et al. [113•], who reported 32% of the variability in BMI accounted for by an MRS, also others, such as Hamilton and colleagues [130], observed that an MRS correlates with adverse health outcomes and accounts for 10% of the variance in BMI. This is similar to observations in adult women where DNA methylation scores roughly explained 10% BMI variance in the population, whilst much less variance was explained in children (1–2%) and young adolescents (3%) [131]. Furthermore, the same study concluded that MRS is a poor marker for future BMI prediction, illustrating the challenges in using MRSs as a meaningful prediction tool in clinical day life so far. However, it has been shown that epigenetic predictors based on DNA methylation at CpG sites are valuable tools in predicting mortality and exposure to certain environmental factors such as to smoking [132]. This is largely corroborated by a recent study showing that MRSs performed better in adult individuals than polygenic risk scores in explaining variance in smoking and BMI [133]. Taken together, there is a potential that MRSs can evolve into useful tools for clinical decision making in the future, although until now there are still conflicting results published. Of note, by increasing the sample sizes, taking into account potential confounders and combining MRSs with polygenic risk scores in the future might help to overcome current obstacles.

## Conclusion

Enormous advances have been made during the last years in identifying genetic and epigenetic loci being involved in the pathophysiology of obesity and related clinical traits. GWAS and EWAS approaches are both by nature hypothesis-free strategies that have proven excellent tools in discovering such novel susceptibility loci and sites. Although for most of the genetic risk variants still the mechanistic circuitry needs to be investigated, important progress has been made for a number of important players.. The use of polygenic risk scores in predicting future BMI or obesity is still in its infancy as a relatively frequent mis-prediction is complicating effective use in clinical settings. Likewise, numerous epigenetic studies have identified novel candidate CpGs and genes conferring changes in DNA methylation. Multiple genes also provide a plausible functional implication in related clinical traits. Construction of methylation risk scores has proven successful for predicting exposure to specific environmental factors such as smoking, but again, its utility in clinical day life is limited for prediction of disease risk. At this stage of research, it seems unlikely so far to use either polygenic or methylation risk scores as a valid clinical prediction tool in the near future. However, by further refining the scores, increasing sample sizes and improving weighting statistic, taking into account typical confounders and combining potentially polygenic risk scores with methylation risk scores may prove successful instruments useful in clinical settings such as predicting future BMI and obesity or predicting successful weight loss in the future.

### Supplementary Information

Below is the link to the electronic supplementary material.

Supplementary file1 (XLSX 2529 KB)
